# Insomnia
*Communicated to the Bristol, Bath, and Somerset Branch of the British Medical Association on 27th April, 1932.


**Published:** 1932

**Authors:** R. G. Gordon

**Affiliations:** Assistant Physician, Royal United Hospital, Bath


### INSOMNIA.*

BY

R. G. Gordon, M.D., D.Sc., F.R.C.P.E.,

*Assistant Physician, Royal United Hospital, Bath.*

If we are to arrive at any satisfactory conception of our subject, we must recognize the important fact that insomnia is a symptom and not a disease. Most people will, of course, readily admit of a reason for not sleeping, but it is only too common to hear people who ought to know better declaring that such and such an event is due to insomnia, and leaving it at that. I refer especially to suicide, which is so often attributed to insomnia, apparently to the satisfaction of coroners' juries and all others concerned. No doubt the symptom of insomnia may have accompanied the emotional state which led to suicide, and even intensified the trouble by the process of a vicious circle, so that it was the last straw which tipped the balance towards self-destruction; but it would be just as sensible to be satisfied with the statement that cough led to night sweats, and to neglect the underlying tuberculous infection which is responsible for both of these symptoms. A further danger of this conception is that both patient and doctor are all too apt to be satisfied if the symptom is treated and no attempt is made to go further. This leads to a widespread exhibition of hypnotic drugs, with

* Communicated to the Bristol, Bath, and Somerset Branch of the British Medical Association on 27th April, 1932.

M

or without the intervention of the medical man. I should be the last to deny the enormous value of such drugs, but they must be intelligently used.

It is often necessary for the success of ultimate treatment and desirable in the interests of humanity to remove such a distressing symptom as that of insomnia as soon and as rapidly as possible; but we should remember the lessons so insisted upon by our teachers, that the greatest circumspection should be used in the employment of analgesics for abdominal pain until a confident diagnosis has been made. So a corresponding therapeutic discretion must be used in the treatment of insomnia, and a diagnosis of the cause of this symptom must be insisted upon in order to lead to a cure of the disease of which this may be the most outstanding manifestation. To me it was a somewhat disquieting experience to hear more than one speaker at a recent discussion on suicide state that all that was necessary to prevent suicide was to treat the insomnia, and I confess I had a mental vision of the unfortunate patient eventually using the bottle of veronal tablets to cure him of his insomnia and his life at one and the same moment.

There can be no clear understanding of the nature of insomnia—the lack of sleep—unless we first understand the nature of sleep. To this extremely difficult question, "What is sleep?" I believe we have at last been given an answer by Pawlow and his assistants, which is at least pragmatically useful. During twenty-five years of increasingly exact experiments on the conditioned reflexes of dogs it was often found that the experiments were vitiated by drowsiness of the animals, which frequently passed into a deep sleep. This is not the place to enter into the story of the conditioned reflexes, but certain experimental conclusions arrived at by Pawlow must be stated in order to understand this phenomenon. These are :—

1. The conditioned reflex is a cortical reaction.

2. Unlike the reflexes of the spinal cord and brain stem, these cortical reflexes are quickly susceptible to fatigue.

3. A conditioned reflex which is frequently repeated becomes "inhibited."

4. This "inhibition" or cessation of response is a symptom of fatigue, and is functionally useful in protecting the delicate cortical nerve cells from serious damage.

5. If an area is inhibited in this way the inhibition tends to spread to adjacent areas of the cortex, unless a fresh stimulus stops this advance of the inhibition. Thus, suppose an inhibition to be spreading from the occipital cortex from the fatigue of a visual reflex, this may be checked by the excitation of the temporal cortex by an auditory stimulus.

6. If unchecked, the inhibition may spread until it involves the whole cortex and even certain of the lower centres. Such widespread internal inhibition is sleep.

7. It was found that sleep intervened in the case of experimental animals under two conditions: (*a*) When a monotonous stimulus was repeated without the intervention of any extraneous stimulus. Here there was an unchecked spread of inhibition which we may compare with the drowsiness and sleep induced by any monotonous occupation, such as that of listening to scientific papers, or the effect of such measures as the counting of sheep going through a gate. (*b*) When a multitude of simultaneous stimuli were all referred to the cortex in the form of conditioned reflexes. This results in a general fatigue sufficient to induce such a widespread cortical inhibition as to constitute sleep. We may compare this to the sudden deep sleep which follows extreme fatigue, or—better still—to the phenomenon often seen in children and puppies of sudden deep sleep suddenly supervening on wild and vigorous play.

This conception of the nature of sleep explains quite a number of problems which are otherwise somewhat baffling. If sleep is an internal inhibition as a result of the fatigue of afferent impulses reaching the cortex, and if such impulses are continually impinging on the cortex, then the inhibition will from time to time be sufficiently widespread to constitute sleep, providing any stimulus is not of such intensity as to check the spread of such inhibition. Hence we find that in normal health sleep is periodic, and ensues without the subject being conscious of any excessive fatigue.

Since all afferent stimuli proceed by way of the thalamic region, disturbances to this part of the brain, such as occur in encephalitis lethargica, may well disturb the normal rhythm of sleep, either by increasing the inhibition, by blocking these afferent stimuli, or interrupting it by fresh stimuli, initiated from the inflamed area. So far, then, and only so far, may we speak of a sleep centre, for sleep involves the whole cortex, but the paths to that cortex may pass through a very restricted area, and thus a restricted lesion may affect a wide expanse of nervous tissue, just as the bombing of an important railway junction disorganized the transport to a long line of battle front.

It is clear that the extent of inhibition of the cortex which constitutes sleep varies from time to time and from individual to individual. Some people sleep like a log, others lightly; some dream, some do not. A dream is due to the stimulus of a pattern which disturbs the general inhibition sufficiently to allow the subject to be aware of the dream, and yet not sufficiently to allow of awakening, though a continuance and further spread of this excitation may wake up the subject. The nature of the state of being awake is obscure, but seems to involve, amongst other things, a correct reference in time and space in respect of the ego and the environment. In the dream there is no correct reference in time and space. A dream, however, may involve such an extent of stimulation that organized motion in the form of somnambulism is possible without the patient being awake. It is obvious, therefore, that much has still to be learnt as to the exact functions of the cortex, and while we may be reasonably certain from Pawlow's work that sleep involves a quantitative rather than a qualitative inhibition, we are still ignorant of the limits of that quantity.

From this we may conclude that the question presented to us by a patient who does not sleep is this: What is preventing the normal spread of internal inhibition throughout the cortex? The answer is, some abnormal afferent stimulus starting some fresh area of excitation and so interrupting this spread of inhibition.

We may now consider the nature of the various stimuli which arouse fresh centres of excitation in the cortex. The first and perhaps the greatest enemy of sleep is pain. Recent work has suggested that pain is not really a specific sensation, but is due to massive and irregular discharges of afferent stimuli from any sensory nerve ending. Pain is apparently perceived subcortically in the thalamus, the cortex being concerned with its localization and with discrimination as to its severity. The arrival of such stimuli at the thalamus, however, is apt to start impulses in all sorts of directions in the thalamo-cortical radiation, so that sleep is very easily disturbed. If pain is responsible for insomnia, it is our duty to remove the source of this pain, or (if this is not possible) to prevent the painful stimuli from reaching the thalamus. This may be done in a few cases by such measures as a division of the posterior spinal roots, but for this purpose we must rely for the most part on the analgesic drugs, especially the opium derivatives headed by morphia. Morphia is a drug which is very effective as a rule in blocking painful stimuli, and has some general depressing effect on the cortex in ordinary doses; but if insomnia is not due to the intervention of pain, morphia is not likely to be successful as a hypnotic, except in elderly patients, for whom the milder opium preparations prove excellent hypnotics. I mention this simply to point out that we must discriminate in the use of hypnotics, and so far as our knowledge extends use the appropriate remedy for the appropriate occasion.

Apart from pain, which as we have seen is not a specific but a general sensation, we may classify sensory stimuli into Sherrington's three groups.

(i.) *Enteroceptive sensations.*—These come from the various internal organs, and by far the most important of these for our purpose are the circulatory. Cardiac patients are notoriously bad sleepers; the asthenic vagotonic young girl with too low blood pressure and the hypertonic old man with too high blood pressure alike sleep badly. A stimulant may cause the one to sleep and a depressant the other. Exercise to warm the patient up before bed may sometimes be useful, but again may be fatal to all chance of sleep ; but if we keep our principles in mind, it should not be difficult to discriminate which line of treatment is suitable for which patient. Similarly, digestive disturbances, irritability of the bladder or sexual excitation, are all liable to interfere with sleep, and the treatment of this form of insomnia is essentially the correction of the visceral anomaly, hypnotics being of little use unless this is done. In this connection diet may be of some importance, as minor degrees of flatulence are often responsible for insomnia. I would suggest, however, that what is necessary is a study of the patient's personal idiosyncrasies rather than a stereotyped régime, and I cordially agree with Crichton Miller in pointing out that the traditional glass of hot milk before retiring may send the patient to sleep because it is hot and wet, but may easily wake him up again because many people cannot easily digest milk in the recumbent position, and the resultant butyric fermentation blows out their stomachs to the detriment of their slumbers.

(ii.) *Proprioceptive sensations.*—These are the afferent impulses from the muscles and joints and locomotor system generally, and normally serve to record our position in space and the movements which we make through space. These sensations are abnormal in conditions of abnormal muscular tone, and are specially liable to interfere with sleep in such conditions as paralysis agitans and post-encephalitic Parkinsonianism. In such cases the drug which seems to block these aberrant stimuli and at the same time produce a general sedative effect is hyoscine, and in my opinion this is the drug of choice in this particular connection. It is similarly often useful in the exaggerated motor activity of mania.

(iii.) *Exteroceptive sensations.* — These are the sensory impulses which come to us from the environment, whether by way of sight, hearing, smell, taste or common sensation (touch, heat and cold). Sleep is thus difficult in a bright light, a noisy room or in a hard bed, whether the happy hunting-ground of livestock or not. The normal environment in which we seek sleep avoids these extraneous stimuli, but in special circumstances we may have to take special precautions to eliminate these, whether by the exhibition of drugs or by other means.

All the foregoing stimuli, which may set up areas of fresh activity in the cortex and so defeat the spread of inhibition which is sleep, come to the cortex from subcortical levels, but more important even than these are those which arrive by transcortical routes. These are the psychic stimuli, chiefly of an affective or emotional nature, which must always be looked for in the examination of a case of insomnia. These disturbances are by no means always obvious to the physician or even to the patient, and call for considerable acumen in their elucidation. These are of special importance from the point of view of medicinal therapy, for it is often found that drugs, except in "knock-out" doses, have little effect. Shakespeare with his usual perspicacity realized this, for Iago says of the jealous Othello :

> "Not poppy nor mandragora,
> Nor all the drowsy syrups of the world
> Shall ever medicine thee to that sweet sleep
> Which thou ow'dst yesterday."

This is specially the case with morphia, for morphia, except in very large doses, is not a good hypnotic, while it is an excellent analgesic. If pain, therefore, is the sole obstacle to the spread of inhibition morphia is very good therapy for insomnia; but if the obstacle to sleep is mental conflict, then it has to be remembered that the first effect of morphia, and to a less degree of alcohol, is to produce an euphoria in which all conflict is abolished, and a peace which passes his understanding descends upon the fortunate or perhaps unfortunate patient. It is this euphoria which he comes to desire far more than relief from insomnia, and he would far rather remain awake with such peace of mind than sleep without it. It is in this way that habits are made.

In certain of the psychoses, and in some of the more severe types of psychoneuroses, there may be such activity of the cortex, and there may be such a vicious circle of over-fatigue with violent activity that it is absolutely essential that the patient should sleep before anything in the way of treatment can be attempted. Under these circumstances drugs which have a general sedative effect on the cortex, such as paraldehyde, bromides, chloral and the barbiturates (luminal, etc.) are useful. The latter are particularly useful if a mild but prolonged effect is desired, since they are slow in action, but their effect is less transient than other hypnotics.

True cases of exhaustion neuroses and psychoses, though often simulated by anxiety and depressive states, are not so uncommon as some would have us believe. In these, as in the compulsive states, the habit of sleep is lost and must be restored. To my mind, the best drug in these cases is medinal, but this brings up an important point in dealing with hypnotics. As everyone knows, the number of drugs annually produced by optimistic firms as at last the perfect hypnotic are legion, and no doubt everyone has his favourite amongst them, some preferring a suffix or prefix to a Greek word and others to a Latin. The truth is that it does not matter very much which of them is used so long as the physician who prescribes them knows how to use them; and personally I think the successful therapist will be the doctor who can use one or two with discrimination, rather than he who knows the name of half a score.

Anxiety cases and hysterics must be treated very carefully if drugs are to be used. The anxiety-neurotic seeks relief from his conflict before he seeks sleep, and the danger has been referred to in connection with morphia. The hysteric seeks a place in the limelight; and if he, or more often she, can by the symptom of insomnia secure the attendance of the physician to prescribe one drug after another, each more expensive than the last, especially if husband or father is paying, then the symptom is not likely to be given up. The unconscious but none the less real tendency of such patients to develop an undesirable dependence is illustrated when massage is used to induce sleep. In its place this is an extremely valuable remedy for insomnia, since it relaxes tension, redistributes the circulation and provides a monotonous stimulus; but we must remember the rich woman who is indeed sent to sleep by her masseuse, but cannot stir from home without her, lest her affliction, as she is fond of calling it, might return. The advisability of removing a symptom by establishing this permanent parasitism must be carefully considered.

I have endeavoured in this paper to sketch out the field of this rather diffuse subject, and since its object is to open a discussion it does not attempt to fill in any details.

